# The Role of miRNA in Renal Fibrosis Leading to Chronic Kidney Disease

**DOI:** 10.3390/biomedicines11092358

**Published:** 2023-08-23

**Authors:** Anna Gluba-Sagr, Beata Franczyk, Magdalena Rysz-Górzyńska, Janusz Ławiński, Jacek Rysz

**Affiliations:** 1 Department of Nephrology, Hypertension and Family Medicine, Medical University of Lodz, 90-549 Lodz, Poland; 2 Department of Ophthalmology and Visual Rehabilitation, Medical University of Lodz, 90-549 Lodz, Poland; 3 Department of Urology, Institute of Medical Sciences, College of Medical Sciences, University of Rzeszow, 35-055 Rzeszow, Poland

**Keywords:** fibrosis, chronic kidney disease, miRNA

## Abstract

Chronic kidney disease (CKD) is an important health concern that is expected to be the fifth most widespread cause of death worldwide by 2040. The presence of chronic inflammation, oxidative stress, ischemia, etc., stimulates the development and progression of CKD. Tubulointerstitial fibrosis is a common pathomechanism of renal dysfunction, irrespective of the primary origin of renal injury. With time, fibrosis leads to end-stage renal disease (ESRD). Many studies have demonstrated that microRNAs (miRNAs, miRs) are involved in the onset and development of fibrosis and CKD. miRNAs are vital regulators of some pathophysiological processes; therefore, their utility as therapeutic agents in various diseases has been suggested. Several miRNAs were demonstrated to participate in the development and progression of kidney disease. Since renal fibrosis is an important problem in chronic kidney disease, many scientists have focused on the determination of miRNAs associated with kidney fibrosis. In this review, we present the role of several miRNAs in renal fibrosis and the potential pathways involved. However, as well as those mentioned above, other miRs have also been suggested to play a role in this process in CKD. The reports concerning the impact of some miRNAs on fibrosis are conflicting, probably because the expression and regulation of miRNAs occur in a tissue- and even cell-dependent manner. Moreover, different assessment modes and populations have been used. There is a need for large studies and clinical trials to confirm the role of miRs in a clinical setting. miRNAs have great potential; thus, their analysis may improve diagnostic and therapeutic strategies.

## 1. Introduction

Chronic kidney disease (CKD) poses an important health concern, and it is expected that by 2040, it will be the fifth most widespread cause of death worldwide [1]. CKD is defined as “a progressive loss of kidney function with accompanying kidney damage or estimated glomerular filtration rate (eGFR) <60 mL/min/1.73 m^2^ lasting >3 months or longer” [2]. Its incidence in developed countries reaches 10–15% [2,3,4]. Glomerulonephritis, including diabetic nephropathy, IgA nephropathy, and lupus nephritis, as well as hypertension and diabetes mellitus (DM), appear to be the most frequent causes of CKD [5,6]. The development and progression of CKD are also associated with the presence of chronic inflammation, oxidative stress, ischemia, etc. [7]. Regardless of the primary origin of renal injury, tubulointerstitial fibrosis appears to be the final common pathomechanism of renal function loss, leading to end-stage renal disease (ESRD) [8,9,10]. Characteristic features of renal fibrosis involve the deposition of extracellular matrix (ECM) proteins in glomeruli and interstitial regions [11]. Moreover, epithelial–mesenchymal transition (EMT) has been suggested to contribute to renal interstitial fibrosis as a result of enhanced expression of vimentin, fibronectin, and α-SMA, as well as reduced levels of E-cadherin in kidney epithelial cells [12,13,14]. Thus, it appears that antifibrotic therapy might be a therapeutic option to hamper the progression of CKD. Various studies have shown that microRNAs (miRNAs, miRs) are involved in the onset and development of fibrosis and CKD [15,16,17]. miRNAs are small (consisting of approximately 18–25 nucleotides), noncoding, single-stranded RNAs that play a vital role in various biological processes via the regulation of gene expression [5,18,19]. Generally, miRNAs bind to the 3′ untranslated region of the target mRNAs in order to stimulate their degradation or inhibit the translation [20]. miRNAs have been demonstrated to modulate various biological and pathological processes, including proliferation, migration, differentiation, and apoptosis [21,22]. Aberrant expression of miRNA has been found to be associated with pathological conditions, including cancers, inflammatory diseases, as well as metabolic disorders [23,24,25]. Several miRNAs were demonstrated to participate in the development and progression of kidney diseases [26,27,28].

## 2. Renal Fibrosis

**Figure 1:**
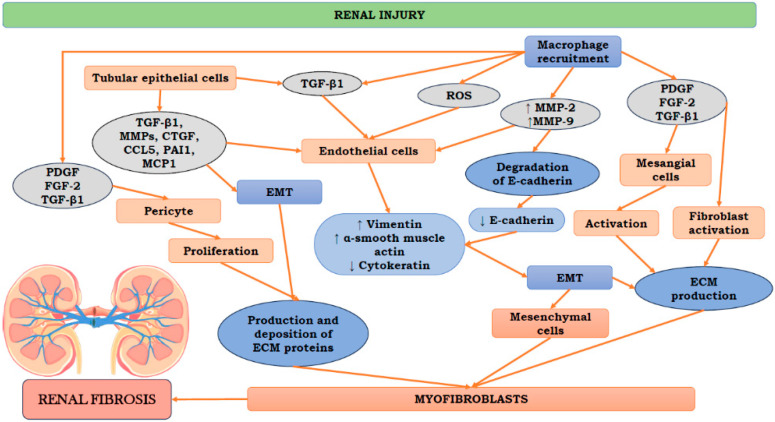
The mechanisms observed during renal fibrosis. ↑ increased; ↓ reduced; CCL5, C-C motif chemokine ligand 5; ECM, extracellular matrix; EMT, epithelial–mesenchymal transition; FGF-2, fibroblast growth factor 2; MCP1, monocyte chemoattractant protein-1; MMP2- matrix metalloproteinase 2; MMP9- matrix metalloproteinase 9; PAI1, plasminogen activator inhibitor-1; PDGF, platelet-derived growth factor; ROS, reactive oxygen species; TGF-β1, transforming growth factor β1.

As mentioned above, renal fibrosis appears to be the common element in almost all progressive kidney diseases [29,30]. Tubulointerstitial fibrosis starts at the early stages of CKD and its degree appears to be critical for further prognosis of individual patients [8]. Unclear boundaries between the cortex and medulla, boosted kidney reflection, and sometimes renal shrinkage are the most common clinical features of renal fibrosis [31,32,33]. Other characteristic features involve the build-up of extracellular matrix (ECM), activation and proliferation of fibroblasts, as well as damage to the tubular epithelium [34,35]. Abnormal tissue regeneration associated with renal fibrosis results in cellular stress and tissue damage [17,36]. Additionally, enhanced tubular cell apoptosis can be observed in the course of renal fibrosis [37]. Zhou et al. suggested that the inhibition of apoptosis in renal tubular epithelial cells might be a promising antifibrotic therapy [38]. The exact pathogenesis of renal fibrosis remains not fully elucidated and there is currently no effective therapy except for lifetime dialysis or kidney transplantation [35,39]. Numerous studies have indicated an important role of epithelial–mesenchymal transition (EMT) of renal tubular epithelial cells in renal fibrosis pathogenesis [40]. The EMT phenomenon was observed for the first time in embryogenesis [41]; however, later studies demonstrated its role in wound healing [42], inflammation [43], cancer progression [44], and fibrosis [45]. The involvement of EMT in fibrogenesis in renal tubular epithelial cells was discovered in a mouse anti-tubular membrane disease model [46]. Further studies have reported that EMT in the kidneys is a complex process comprising a loss of epithelial cell adhesion, damage of the basement membrane, the synthesis of α-SMA, as well as cellular migration and invasion [40,47]. During EMT, injured epithelial cells could serve as a vital source of fibroblasts, favoring kidney fibrosis. According to its definition, EMT is associated with phenotypic and functional alterations related to mesenchymal cells. During renal fibrosis, epithelial cells have been found to migrate from the tubular structure into the interstitium and form a matrix, acting at that time as new fibroblasts [40]. The trans-differentiation of renal tubular epithelial cells into myofibroblasts appears to be a crucial mechanism involved in fibrosis development [48]. This process involves characteristic modification of epithelial cells, loss of tight junctions between cells, and structural impairment of the integrity of the basement membrane [49,50,51]. Moreover, excessive interstitial extracellular matrix (ECM) deposition is observed during EMT [40,52]. Targeting the EMT process, especially the Wnt/β-catenin pathway, seems to be a vital therapeutic strategy for patients with renal fibrosis. The sustained activation of Wnt/β-catenin signaling has been found to significantly accelerate the EMT process [53]. The Wnt/β-catenin pathway is an evolutionarily conserved pathway participating in the formation of nephron; thus, it is quiescent in normal adult kidneys. However, this pathway can be reactivated, e.g., following kidney injury [54,55]. Following the activation of injury-induced β-catenin, it favors the amplification of Wnt signaling [40]. The accumulation of free β-catenin was demonstrated to be associated with epithelial integrity destruction [56]. Therefore, focusing on a blockade of the Wnt/β-catenin signaling pathway may represent a potential treatment strategy for renal fibrosis. The mechanisms observed during renal fibrosis are presented in Figure 1.

Renal fibrosis is associated with elevated synthesis of α-smooth-muscle actin (α-SMA), higher activity of metalloproteinase (MMP2 and MMP9) and extracellular matrix (ECM) in the interstitium, as well as decreased expression of E-cadherin [57]. It is also related to aberrant regulation of some chemokines and growth factors (e.g., transforming growth factor-β1 (TGF-β1), monocyte chemoattractant protein-1 (MCP-1), regulated upon activation, normal T cell expressed and presumably secreted (RANTES), and platelet-derived growth factor (PDGF)), uremic toxins, lipid metabolism factor, as well as the dysfunction of the renin-angiotensin system [58,59,60]. TGF-β1 is a key factor contributing to the development of renal fibrosis since it stimulates both renal tubular epithelial cells’ transformation into myofibroblasts and the deposition of ECM. It upregulates SMA and downregulates E-cadherin protein expression [47]. In vivo studies demonstrated a positive correlation between TGF-β1 expression, exosome secretion, and renal fibrosis [61]. TGF-β1 triggers the Smad signaling pathway [62]. Usually, the stimulation of TGF-β leads to the phosphorylation of Smad2 and Smad3 (R-Smads), the activation of Smad4 (C-Smads), and the modulation of transcription of genes downstream of TGF-β. However, since I-Smads act as the competitors of R-Smads, they hamper phosphorylation of the latter, which results in the inhibition of TGF-β signaling pathways [63]. The activation of Smad3 in the course of TGF-β1-induced renal tubular epithelial cell trans-differentiation was found to correlate with elevated collagen expression, while the higher expression of Smad7 can partly reverse fibrosis development [64,65]. Smad3 has been demonstrated to mediate renal fibrosis via both the downregulation of miR-29 and miR-200 and the upregulation of miR-21 and miR-192. Indeed, the reduction of Smad3-mediated renal fibrosis was observed in mouse models of chronic kidney diseases overexpressing miR-29 and miR-200, or showing decreased expression of miR-21 and miR-192 [66,67]. Some actions of TGF-β1 are associated with the regulation of certain miRs in mesangial cells and podocytes. For example, it was reported to upregulate miR-34a, miR-192, miR-377, and miR-491, as well as to downregulate miR-29 and miR-30 [23,24,68]. It has been suggested that the determination of profibrogenic miRNAs in urine and/or serum could be a promising, noninvasive method of renal fibrosis assessment [8].

Various miRNAs are involved in the process of renal fibrosis. Published results have indicated that some promote fibrosis progression, while others show antifibrotic properties. The roles of several miRNAs in this process are unknown since the outcomes of various studies are conflicting. Nephrectomy (5/6 Nx) is frequently used to mimic advanced-stage nephropathy in order to study the mechanisms underlying CKD progression [69]. The loss of nephrons resulting from 5/6 Nx causes renal tubulointerstitial fibrosis and endocrine capability dysfunction. As described below, 5/6 Nx animal models have been used in many studies. Additionally, unilateral ureteral obstruction (UUO) serves as a model to investigate fibrosis-related mechanisms, which is believed to mimic accelerated human chronic obstructive nephropathy [70]. In this model, obstructed urine flow is the cause.

## 3. Profibrotic miRNA

### 3.1. miR-21

miR-21, which is among the best-studied miRNAs, was suggested to be involved in the regulation of fibroblast proliferation [61,71,72]. The results have demonstrated that miR-21 acts as a profibrotic factor since its upregulation was observed in patients with severe kidney fibrosis [73]. Additionally, animal studies have indicated the involvement of this miRNA in renal fibrosis [74,75]. The upregulation of miR-21 was demonstrated to correlate with the extent of fibrosis and the decline in renal function in patients with diabetic nephropathy [76]. miR-21 was suggested to promote the development of renal fibrosis through the regulation of metabolic pathways engaged in the oxidation of fatty acid and lipids, including the silencing of the PPAR-α axis [77]. In turn, the upregulation of PPAR-α expression in transgenic mice with unilateral ureteral obstruction was associated with diminished accumulation of ECM and decreased renal interstitial fibrosis. In the UUO model, tubule cells containing exosomal miR-21 activated renal fibroblasts via the PTEN/Akt pathway [61]. The results of another study also demonstrated that miR-21 levels in renal tissues were important for the development of renal fibrosis [78]. According to other studies, miR-21 can affect the expression of several metalloproteinases (MMPs) via the downregulation of PTEN during TGF-β1-induced EMT [79]. MMP-2 appears to play role in renal interstitial fibrosis [80]. Over the course of kidney injury, miR-21 has been found to downregulate Mpv17-like protein, which regulates reactive oxygen species (ROS) generation, thus reducing the protection against mitochondrial oxidative stress, apoptosis, and renal fibrosis [77,81]. Moreover, in the kidney, miR-21 stimulates extracellular signal-regulated kinases (ERK) signaling, including ERK1/2 and TGF-β/Smad pathways, which appear to be involved in the development of renal fibrosis [82]. The inhibition of miR-21 protected against cyclosporine A-induced activation of Akt and the expression of the EMT gene, but also limited the activation of profibrotic genes in human podocytes and tubular cells in IgA nephropathy [83,84]. miR-21 regulates TGF-β signaling, but TGF-β1 can also trigger the expression of miR-21 via Smad signaling [85]. Numerous studies have confirmed the role of TGF-β in renal fibrosis [76,81]. TGF-β1-stimulated induction of miR-21 occurs at the posttranscriptional level since no rise in pri-miR-21 is observed [86]. Another study revealed that the regulation of miR-21 via TGF-β and bone morphogenetic protein (BMP) signaling was associated with the binding of Smad3 to stem regions of the target pri-miRNA and subsequent modulation of its expression in response to TGF-β stimulation [87,88]. An alternative mechanism of miR-21-induced renal fibrosis may involve the decrease of dimethylarginine dimethylaminohydrolase 1 (DDAH1) activity/expression. The upregulation of this miRNA was found to increase the level of asymmetric dimethylarginine (ADMA), which is an endogenous inhibitor of nitric oxide synthase (NOS), thus diminishing the production of NO [89]. Moreover, the use of anti-miR-21 was found to diminish renal fibrosis and injury in animal models of acute kidney injury and Alport syndrome [77]. The administration of anti-miR-21 oligonucleotides to mice with Alport disease triggered beneficial effects as it decreased interstitial fibrosis and glomerulosclerosis, reduced inflammation and tubular injury, and prolonged survival [90]. However, in a study conducted by Fujii et al. [7], miR-21 played a nephroprotective role, which was suggested to be related to its anti-inflammatory properties. According to the authors, reduced expression of miR-21 in CKD could be associated with enhanced podocyte loss [91]. Since the loss of podocytes can lead to glomerulosclerosis, miR-21 may protect against glomerular injury [92]. Another hypothesis states that elevated levels of this miRNA could diminish the level of inflammatory cytokines via silencing of phosphatase and tensin homolog deleted on chromosome ten (PTEN) and programmed cell death protein 4 (PDCD4), thus limiting the inflammatory state.

### 3.2. miR-34a

Enhanced expression of miR-34a was suggested to play a vital role in renal fibrosis development. Liu et al. [39] demonstrated that miR-34a contributed to renal fibrosis via the downregulation of Klotho. miR-34a was found to directly bind to the 3′ UTR of Klotho mRNA. According to other reports, Klotho, mostly expressed in renal tubular epithelial cells, protects the kidneys against acute and chronic injury, while lower levels were observed in patients with renal diseases [93,94,95,96]. Decreased levels of Klotho correlated with hastened profibrotic phenotype transition in tubular epithelial cells and increased proliferation of fibroblasts [93,97]. In turn, the inhibition of miR-34a-mediated Klotho in tubular epithelial cells hampered the progression of renal fibrosis in a mouse model with UUO and the Adriamycin (ADR) nephropathy. Moreover, miR-34 deficiency in a miR-34a^−/−^ mouse model improved renal fibrosis after obstructive injury. Beneficial effects related to Klotho overexpression are due to the suppression of profibrotic signaling pathways, such as Wnt, TGF-β1, and FGF2 [93,97]. The upregulation of miR-34a may be involved in the feedback loop between the activation of TGF-β1 and the Klotho decrease in the progression of renal fibrosis [39]. However, according to other studies, abnormal expression of miR-34a is not the only mechanism for decreasing Klotho levels. Upregulated expression of miR-34a-5p in renal tissues of patients with type 2 diabetes mellitus (T2DM) and mice with diabetic nephropathy (DN) was associated with the reduced expression of the Ski-related novel gene (SnoN), promotion of EMT, and more severe fibrosis [17]. Other studies suggested that downregulation of the expression of silent information regulator 1 (SIRT1) (involved in tubulointerstitial fibrosis in DN) is responsible for miR-34a-5p-related fibrosis [98,99].

### 3.3. miR-130a-3p

miR-130a-3p was also suggested to exert profibrotic effects. The upregulation of this miRNA was observed in CKD and other fibrotic diseases [100]. miR-130a-3p may be involved in the modulation of EMT and fibrosis in TGF-β1-induced renal tubular epithelial cells [35]. The inhibition of miR-130a-3p expression was found to be associated with a considerable reduction in the level of α-SMA and vimentin mRNA and protein, as well as the upregulation of E-cadherin expression. Moreover, such inhibition translated into significantly decreased TGF-β1 and p-Smad2/3 protein levels and increased Smad7 levels, which implies the activation of an inhibitory signal. An in vitro study provided evidence for the important role of miR-130a-3p in the regulation of Smads involved in the TGF-β signaling pathway during renal fibrosis development. The upregulation of miR-130a-3p was demonstrated to be associated with fibrosis via the activation of the TGF-β1/Smad pathway and the targeting of SnoN. High expression of miR-130a-3p translates into a reduced SnoN level [35]. The results confirmed that miR-130a-3p blocked the expression of the SnoN gene at both the transcriptional and posttranscriptional levels [35]. In turn, SnoN can inhibit the transmission of the TGF-β1/Smad signal, and its overexpression may partly reverse the effects related to TGF-β1. The SnoN gene contains a miR-130a-3p-targeted sequence in the 3′UTR, which confirms that it is the target gene of miR-130a-3p. Thus, the blocking of miR-130a-3p was suggested to be able to protect against renal fibrosis in vitro.

### 3.4. miR-132

miR-132 is organized in tandem with miR-212 on chromosome 17 and they are transcribed together under the regulation of the cAMP response element binding protein (CREB) [101,102]. CREB expression is controlled by angiotensin II (ANG II) [101]. In the course of progressive renal fibrosis, perivascular cells were found to be the source of the substantial amount of α-smooth-muscle actin (α-SMA)-positive myofibroblasts [103]. Bijkerk et al. [103] observed that in UUO mice, the expression of miR-132 was 21 times higher during pericyte-to-myofibroblast formation. Moreover, the silencing of this miRNA during obstruction was associated with diminished collagen deposition as well as tubular apoptosis. In vitro analysis confirmed that miR-132 played a rate-limiting role in interstitial myofibroblast proliferation. However, the use of antagomir-132 did not disturb the reparative proliferation of tubular epithelial cells [103]. In turn, the analysis of pathways associated with the actions of miRNAs- demonstrated that miR-132 regulated genes involved in TGF-β signaling (Smad2/Smad3), STAT3/ERK pathways, and Foxo3/p300 (cell proliferation). According to the authors, the ability of miR-132 to selectively reduce myofibroblast proliferation and limit the progression of renal fibrosis could be used to develop antifibrotic therapy.

### 3.5. miR-199a

miR-199a and miR-214 are co-transcribed on a single long-noncoding RNA (lncRNA), which is located within the intron of dynamin-3 gene on the complementary strand, and they are produced from a single molecule, pre-miR-199p [81,104]. The expression of both these miRNAs is enhanced in a hypoxic state via HIF-1α, but also by the activation of the TWIST transcription factor. The role of miR-199a has not yet been determined; however, the results of studies involving non-renal tissues suggested that it regulates the migration of cells and matrix deposition [104]. Cardenas et al. [105] demonstrated higher renal expression of miR-199a-5p in the UUO mouse model of kidney fibrosis. Moreover, they found that compared to miR-199a-3p, miR-199a-5p more efficiently induced TGF-β-mediated lung fibroblast activation via targeting caveolae and decreasing caveolin-1 levels. Another study pointed to the membrane protein Klotho as a target of miR-199a-5p in rat mesangial cells [106]. miR-199a-5p-related inhibition of Klotho expression was demonstrated to enhance the expression of inflammatory and profibrotic genes due to the stimulation of Toll-like receptor 4 (TLR4)/NF-κB p65/NGAL signaling.

### 3.6. miR-214

Many studies have demonstrated increased expression of miR-214 in animal models and in humans with kidney disease [107]. Its expression has been detected in glomeruli, renal tubules, as well as infiltrating immune cells [108]. In one study, the profibrotic activities of miR-214 were suggested to be independent of TGF-β signaling [108]. The reduction of miR-214 protected against fibrosis in a UUO mouse model. miR-214 was found to act via the endogenous Akt signaling pathway inhibitor PTEN [109]. This finding was confirmed by in vitro studies. The inhibition of this miRNA was associated with partial restoration of PTEN protein levels in the culture of human mesangial cells.

### 3.7. miR-433

The gene miR-433, belonging to the DLK1-DIO3 miRNA cluster, is localized at chromosomal region 14q32.2. It acts as a tumor suppressor in various human cancers [81]. Expression of miR-433 was found to be regulated at the epigenetic level. According to one study, the use of the DNA methylation inhibitor 5-aza-2′-deoxycytidine (5-aza-CdR) leads to increased expression of this miRNA [110]. The results have revealed that miR-433 is involved in profibrotic activity. Its upregulation was demonstrated in renal and cardiac fibrosis [111,112]. In an animal model, unilateral ureteral obstruction was associated with increased levels of this miRNA. In turn, knockdown of this miRNA was demonstrated to reduce the onset and progression of renal fibrosis in a UUO model [112]. miR-433 activates the transforming growth factor-β (TGF-β)/Smad3-Azin1 signaling pathway, thus promoting renal fibrosis [112]. miR-433 was demonstrated to trigger a positive feedback loop, intensifying TGF-β/Smad3 signaling. Antizyme inhibitor 1 (Azin1) is a target gene of miR-433. Li et al. [112] found that the overexpression of miR-433 was associated with reduced expression of Azin1 and consequent promotion of TGF-β signaling and fibrosis.

### 3.8. miR299a-5p

miR299a-5p was found to be involved in the regulation of renal fibrosis [113]. Following the upregulation by TGF-β1, the production of the antifibrotic protein follistatin (FST) is reduced, thus launching the profibrotic response. FST counteracts the profibrotic and pro-inflammatory activity of some TGF-β superfamily members, especially activins [114]. In turn, the inhibition of miR299a-5p diminishes renal fibrosis and prevents CKD progression. The impact of miR299a-5p on FST 3′UTR stability translates into decreased expression of transcripts. Mehta et al. [113] observed that a weakened TGF-β1 profibrotic response can be reversed by the overexpression of miR299a-5p and related activin A activity and Smad3 signaling [115,116]. Enhanced expression of miR299a-5p has been associated with fibrosis of various organs, including the lungs, liver, and heart [117,118,119]. Data on the role of miR299a-5p in renal fibrosis are sparse. Mehta et al. [113] demonstrated increased levels of miR299a-5p in glomeruli and tubules in a CKD model. In their study, the inhibition of miR299a-5p was associated with the rise in FST expression in the kidneys of CKD mice and higher protection against renal fibrosis. Moreover, the authors also observed functional improvements involving the increase in GFR and decrease in albuminuria resulting from miR299a-5p inhibition. However, no significant increase in serum FST levels was reported. Interestingly, FST neutralization targets other than activin A (e.g., myostatin) were found to promote fibrosis in skeletal muscles via a Smad3-related mechanism [120]. It has been suggested that an increase in FST expression/miR299a-5p inhibition may also diminish renal fibrosis via epigenetic regulation of histone deacetylase (HDAC)-mediated expression of profibrotic proteins. This enzyme catalyzes protein deacetylation, which is involved in the modulation of physiological and pathological expression of genes. Liu et al. [121] found that the inhibition of HDAC suppressed the progression of renal fibrosis in various animal models. The analysis of the impact of miR299a-5p inhibition on processes contributing to the development of kidney fibrosis, including macrophage and T-cell infiltration, failed to show positive results [113]. However, miR299a-5p inhibition was demonstrated to hamper cell proliferation in CKD. Moreover, Mehta et al. [113] observed the protection against CKD-related glomerular podocyte and endothelial cell loss following miR299a-5p inhibition, which can be associated with Smad3 pathway inhibition. However, the authors also suggested that the increased expression of FST may be related to lower renal protection, possibly due to the excessive reduction of oxidative species that are necessary for standard cellular signaling [122].

## 4. Antifibrotic miRNAs

Some miRNAs, including hsa-miR-23b, hsa-miR-29, hsa-miR-30, and hsa-miR-192, have been suggested to exert inhibitory effects on kidney fibrosis, as discussed below.

### 4.1. miR-29 Family

Several miRNAs, including the miR-29 family (miR-29a, miR-29b, and miR-29c), have been demonstrated to act as antifibrotic factors in CKD [123]. Despite having a common seed sequence, these miRNA are encoded by distinct genomic loci [81]. They target various fibrosis-related genes, such as collagen I and IV, elastin, integrin-β1, laminins, and fibrillin [124,125]. Yu et al. [126] provided evidence for an antifibrotic effect of miR-29, since they demonstrated downregulation of hsa-miR-29-5p expression in the focal segmental glomerulosclerosis (FSGS) and DN groups compared to the control group. miR-29 was demonstrated to be capable of decreasing the expression of approximately 20 various genes related to collagen formation. An in vitro study showed that knockdown of miR-29 was associated with higher TGF-β-triggered expression of collagens I and III by renal tubular cells [127]. In patients with CKD, the levels of miR-29 were found to be decreased [125]. Their lower levels correlated with enhanced expression of extracellular matrix (ECM) proteins, including collagen. It has been suggested that beneficial effects of miR-29 involve the suppression of matrix accumulation and the promotion of cell death, provided that it is profibrogenic cells that undergo apoptosis [81,128]. The antifibrotic effects of miR-29 have been demonstrated in animal models. Wild-type mice with progressive renal fibrosis in obstructive nephropathy were found to have decreased expression of miR-29 [127]. In turn, Smad3 knockout mice had elevated expression of this miRNA. The experiments with cultured fibroblasts and tubular epithelial cells revealed that Smad3 can bind to the promoter of miR-29, leading to its TGF-β-induced downregulation. Moreover, Qin et al. [127] demonstrated that miR-29 acted as a downstream inhibitor of TGF-β/Smad3-mediated fibrosis. The administration of miR-29b, preceding the induction of obstructive nephropathy or after it, was found to inhibit progressive renal fibrosis [127]. Reduced expression of miR-29c markedly enhanced interstitial fibrosis, tropomyosin 1α protein levels, and collagen type IIα1 (col2a1) in kidneys of rats subjected to 5/6 nephrectomy, compared to a sham control [129]. Similar findings were seen in the kidneys of patients with IgA nephropathy. In contrast, the knockdown of HIF-1α or HIF-2α considerably reduced the upregulation of miR-29c expression. Thus, it appears that these two factors contribute to the modulation of miR-29c expression in the kidneys [129]. The downregulation of the miR-29 family is also associated with TGF-β [130].

### 4.2. miR-27b-3p

Bai et al. [131] observed that the overexpression of miR-27b-3p strongly repressed TGF-β1-induced EMT through the downregulation of α-SMA, fibronectin, collagen III, and vimentin in cell cultures. Moreover, increased expression of this miRNA reduced the expression of α-SMA and collagen III in UUO mice, thus hampering renal fibrosis. These findings suggest that increased expression of miR-27b-3p limits renal fibrosis (in vitro and in vivo) through EMT inhibition. The study aiming at the identification of a potential target gene of miR-27b-3p pointed to STAT1, which is involved in the inflammatory response and tumorigenesis [132,133]. The evidence for a role of STAT1 in fibrosis was provided by Zhang et al. [134], who showed that inhibition of this transcription factor suppressed the progress of liver fibrosis. Other studies have suggested that the activation of STAT could stimulate EMT, trigger Fas signaling, and promote renal damage [135,136,137]. TGF-β1 significantly promoted the expressions of p-STAT1 and STAT1 in HK-2 cell cultures, while overexpression of miR-27b-3p reduced the expressions of p-STAT1 and STAT1 [131]. In addition, in the model of UUO kidneys, enhanced expression of miR-27b-3p blocked the expression of p-STAT1 and STAT1 [131]. Moreover, overexpression of miR-27b-3p was found to inhibit TGF-β1-induced and Fas-mediated apoptosis in human kidney 2 cells through the downregulation of active caspase 3, Fas, and active caspase 8 [131]. Disturbed regulation of apoptosis and EMT appears to play a crucial role in renal fibrosis [138]. Both apoptosis and EMT were demonstrated to be involved in miR-27b-3p-mediated regulation in renal fibrosis.

### 4.3. miR-30 Family

The miR-30 family (miR-30a, -30b, -30c, -30d, and -30e) has been suggested to exert antifibrotic effects [123,139]. One study indicated that miR-30 could limit the production of ECM protein by renal tubular epithelial cells, as well as inhibit phenotypic modifications via targeting the mitochondrial uncoupling protein 2 [140]. In turn, Li et al. [141] reported that miR-30 could limit UUO-induced renal fibrosis via targeting Sox9. The miR-30 family can also limit phenotype changes of ECM via the downregulation of mitochondrial uncoupling protein 2 (UCP2) [140]. UCP2 is a direct target of miR-30e. In a UUO mouse model, UCP2 was found to be increased, while inhibition of this protein limited UUO-triggered renal fibrosis. The expression of UCP2 was found to be stimulated by TGF-β1. The overexpression of UCP2 translated into enhanced TGF-β1-induced epithelial-to-mesenchymal transition. Such transition was hampered by a miR-30e mimic. As well as the aforementioned molecules, miR-30 also regulates the expression of connective tissue growth factor (CTGF), which participates in renal fibrosis. The expression of miR-30 was demonstrated to inversely correlate with the amount of CTGF [142]. According to various studies, the increased expression of miR-30 diminished the level of this factor and limited the production of collagens. Downregulation of miR-30e expression in diabetic nephropathy was associated with enhanced EMT and reduced proliferation of renal tubule epithelial cells. In turn, the upregulation of this miRNA resulted in lowered collagen I, vimentin, fibronectin, and alpha-smooth-muscle actin (α-SMA) expression, as well as increased E-cadherin expression. These findings suggest that miR-30e may exert reno-protective effects [143].

### 4.4. hsa-miR-3607-3p and hsa-miR-4709-3p

A study of renal biopsy specimens collected from patients with 3 different pathological types of CKD and analyzed using microarray revealed 40 upregulated miRNAs and 76 downregulated miRNAs [126]. Most of these miRNA have already been described earlier; however, differential expression of two novel miRNAs—hsa-miR-3607-3p and hsa-miR-4709-3p—has also been demonstrated in human biopsy samples and a UUO fibrosis model. The results of functional analyses suggested their involvement in the CKD-related fibrosis process [126]. miR-3607-3p was found to be involved in the regulation of cell cycle and T-cell activation [144]. Target genes of hsa-miR-3607-3p involved in the regulation of the actin cytoskeleton have been found to be upregulated in the fibrotic process [126]. In turn, hsa-miR-4709-3p was demonstrated to have an impact on the mTOR signaling pathway, which is, among others, involved in the development of diabetic nephropathy [145]. A recent study provided evidence for the involvement of EMT in the development of diabetic nephropathy [146]. In the course of EMT, alterations in cell shape, higher motility resulting from increased collagen production, and a loss of polarity are observed [146]. Yu et al. [126] demonstrated that both hsa-miR-3607-3p and hsa-miR-4709-3p participate in the kidney fibrosis process. hsa-miR-4709-3p stimulated actin fibers’ assembly and cell motility, while hsa-miR-3607-3p exerted the opposite effect. The authors also observed lower hsa-miR-3607-3p levels in fibrotic kidneys. ITGB8 was found to be a target of hsa-miR-3607-3p. The upregulation of ITGB8 was suggested to be associated with continuous activation of TGF-β signaling and renal fibrosis [126]. In turn, calmodulin 3 (CALM3) was proposed as a hsa-miR-4709-3p target. The potential role of hsa-miR-4709-3p and CALM3 in kidney fibrosis may be related to changes in intracellular calcium levels.

### 4.5. miR-671-5p

The information on the role of miR-671-5p in fibrosis is sparse. The results of a study conducted by Yu et al. [147] showed that the overexpression of miR-671-5p was associated with higher expression of many fibrosis-related proteins, including collagen I, α-smooth-muscle actin (α-SMA), and fibronectin. miR-671-5p stimulated the expression of transforming growth factor β1 (TGF-β1) and connective tissue growth factor (CTGF) in 5/6Nx mice. The analysis of fibrotic lesions in the 5/6Nx kidneys (triggered by the overexpression of miR-671-5p) revealed enhanced deposition of fibronectin in kidneys [147]. Substantial collagens’ deposition in the kidneys following 5/6Nx was found to be aggravated by the overexpression of miR-671-5p. These results may provide evidence for the impact of miR-671-5p on the growth of renal fibrotic lesions in 5/6Nx mice in vivo. miR-671-5p exacerbated podocyte injury, glomerulosclerosis, and renal fibrosis in 5/6Nx mice [147]. In turn, miR-671-5p antagomir ameliorated these changes [147]. Secondary to the mitigation of podocyte injury and proteinuria, inhibition of miR-671-5p was suggested to decrease kidney interstitial fibrosis. The inhibition of miR-671-5p may have direct beneficial effects on tubular epithelial cells. Finally, the inhibition of miR-671-5p could become a promising approach for the development of therapeutics to treat proteinuric CKDs.

## 5. miRNAs with Controversial Roles in Renal Fibrosis

Some miRNAs have been demonstrated to be involved in renal fibrosis; however, the reports on their pro- or anti-fibrotic effects remain controversial, as discussed below.

### 5.1. miR-192 Family

The gene encoding miR-192 is highly expressed in normal kidneys [81,148]. Both miR-192 and miR-194-2 are located on the same chromosome and are co-transcribed in the shared precursor pri-miR-192/194; thus, they are regulated as a joint transcriptional unit [148,149]. Jenkins et al. [149] found the presence of hepatocyte nuclear factor (HNF) and p53 binding sites within the miR-194-2/192 promoter region necessary for constitutive activity. TGF-β1, acting via an Alk5-dependent mechanism, was demonstrated to reduce HNF binding to this promoter site. Moreover, HNF-1 knockdown limited the expression of mature miR-192 and miR-194 [149]. Furthermore, in human proximal tubular cells, the expression of miR-192 was downregulated by TGF-β1. In turn, enhanced expression of miR-192 was associated with limited expression of Zeb1 and Zeb2 and antifibrotic effects. Krupa et al. [150] found that enhanced fibrosis and a lower estimated glomerular filtration rate were associated with reduced miR-192 in kidney tissues collected from patients with advanced diabetic nephropathy. It has been suggested that the severity of diabetes mellitus may affect the functional role of miRNA-192. However, several other studies have indicated profibrotic actions of miR-192 in renal mesangial and tubular cells [130,151,152]. Kato et al. [152] reported that after the induction, miR-192 targeted and downregulated zinc finger E-box binding homeobox 1/2 (Zeb1/2) expression in order to enhance collagen expression and renal fibrosis. In renal mesangial cells, the levels of TGF-β1 were found to be upregulated by amplifying circuits involving miR-192 or miR-200b/c and TGF-β1 [153]. Kato et al. [153] also observed that the use of miR-192 inhibitors reduced the expression of miR-200b/c, Col1a2, Col4a1, and TGF-β1 in mice mesangial cells and the renal cortex. Such circuits were suggested to accelerate the progression of CKD. Moreover, another study demonstrated the amelioration of renal fibrosis following the blockage of miR-192 in diabetic mice [67]. Chung et al. [151] observed that the deletion of Smad7 translated into a higher expression of miR-192, greater Smad signaling, and consequent fibrosis development in obstructive kidney disease. In turn, the overexpression of Smad7 exerted the opposite effects. Similar to miR-21, miR-192 also favors renal fibrosis via increasing TGF-β signaling. The authors also found that TGF-β1-induced miR-192 expression was mediated by Smad3, but not Smad2. Finally, they reported that the addition of the miR-192 inhibitor blocked TGF-β1-triggered expression of the collagen matrix, while the overexpression exerted a stimulating effect [151]. The conflicting results of the aforementioned studies regarding the role of miR-192 in renal fibrosis may stem from the use of different animal and cell culture models, the various stages of disease, and the conditions of the experiments. For example, HNF expression is limited to the tubular compartment and lacking in renal mesangial cells and podocytes, which may explain differences in cell-specific miR-192 regulation [154].

### 5.2. miR-200 Family

The miR-200 family is involved in the maintenance of epithelial cell differentiation [81]. Members of this family: miR-200a, -200b, -200c, -141, and -429, are encoded by two separate genomic loci on chromosome 1. All these miRNAs have seed sequences which enable the recognition of the 3′-UTR sequence within the target transcript of the regulated genes [81]. The seed sequences for miR-200b, miR-200c, and miR-429 (group 1), as well as miR-200a and miR-141 (group 2), differ; thus, these two groups do not share the same mRNA target [155]. The results concerning the role of the miR-200 family in renal fibrosis are conflicting. It has been suggested that miR-200 may be involved in the protection against epithelial-to-mesenchymal transition, and consequently, against renal fibrosis [155]. The analysis of HK-2 cell culture confirmed that miR-200b inhibited TGF-β1-induced EMT through the reduction of fibronectin mRNA (direct targeting) and protein levels and the increase of E-cadherin (due to lower Zeb1 and Zeb2 expression) [156]. The suppression of TGF-β1-induced EMT was independent of TGF-β1-induced phospho-Smad2/3 and phospho-p38 and p42/44 signaling. One of the studies reported that miR-200a also suppressed TGF-β2 expression, thus preventing the development of renal fibrosis [157]. Moreover, the miR-200 family and miR-205 were found to be downregulated during TGF-β1-mediated EMT in renal epithelial cells, while increased expression of the miR-200 family sufficiently prevented EMT [158]. However, in another study, higher expression of miR-200 family members translated into an increased expression of collagen and enhanced renal fibrosis [153]. It was observed that the upregulation of TGF-β1 levels by miR-200b/c formed an autoregulatory loop, leading to the intensification of TGF-β1 signaling in chronic renal fibrotic diseases [153]. According to the authors, TGF-β1-induced expression of miR-192 can trigger the miR-200 family, which eventually results in Akt activation, hypertrophy, and fibrosis in mesangial cells.

A summary of the roles of the described miRNA in renal fibrosis is presented in Table 1.

## 6. Therapeutic Use of miRNA-Based Therapies in Kidney Disease

The application of miRNAs as therapeutics is not well studied due to associated obstacles that still need to be overcome. The therapeutic potential of miRNAs has been analyzed in a few preclinical studies, mostly performed in rodents or cell cultures in various kidney diseases. To study the impact of miRNAs, various techniques have been developed. Endogenous miRNA can be inhibited by the introduction of anti-miRNA oligonucleotides, designed to target pri-miRNA, pre-miRNA, or mature miRNA in order to sequester endogenous miRNA or remove it [159]. Tandem repeats of a sequence that display perfect complementarity to the miRNA (erasers) can be used to silence the miRNA of interest. RNAs are enormously vulnerable to ribonuclease-mediated degradation in cellular and extracellular environments. To avoid quick decay of miRNAs, several modifications, including replacement of the phosphodiester backbone with a phosphorothioate backbone, peptide nucleic acid modification, and the ribose 2′-OH group, have been introduced to increase the RNA stability in vivo [160,161]. Modifications such as 2′O-methoxyethylphosphorothioate and 2′ fluoro substitutions have been shown to be associated with specific and strong silencing of the miRNA of interest [162]. The locked nucleic acid (LNA)-based approach is also used to silence miR-21. Various double-stranded synthetic oligonucleotides that mimic the function of endogenous miRNAs, but display improved stability and chemical modifications to enhance their effective delivery to target cells, have been tested in various preclinical and clinical studies [163]. In these studies, miRNAs, antagonists of miRNAs (antagomirs), or locked nucleic acid (LNA) derivatives of miRNAs are directly administrated in the form of native nucleotides, nucleotides contained in extracellular micro-vesicles, or manufactured nanoparticles [164,165]. Apart from miRNA antagonists, miRNA-mimics are also used. These synthetic double-stranded precursor miRNA molecules trigger therapeutical overexpression of the target miRNA.

The study of the effect of the miR-132 antagomir in mice with unilateral ureteral obstruction demonstrated that it decreased the number of interstitial myofibroblasts [103]. In turn, transfection of HK cells with miR-294/miR-133 mimics was found to considerably hamper the production of α-SMA, reverse the transformation to a myofibroblast phenotype, probably due to the inhibition of SMAD2/3 and ERK1/2 phosphorylation, and subsequently, prevent TGF-β1-induced EMT [164]. The results of another animal study provided evidence for the beneficial role of miR-200 since it demonstrated that the delivery of the miR-200b precursor improved fibrosis in UUO mice [166]. In another study, early as well as delayed administration of treatment with locked nucleic acid (LNA)-anti-miR-150 to mice that developed tubulointerstitial fibrosis following the injection of folic acid obliterated the increases in renal IFN-γ, IL-6, and TNF-α mRNAs [167]. This study revealed that if LNA-anti-miR-150 was injected (twice per week, for four weeks) prior to or after tubular injury, it was capable of reversing renal inflammation and fibrosis. Moreover, the miR-150 antagonist reversed the upregulation of profibrotic genes encoding α-smooth-muscle actin, fibronectin (FN), and collagen 1 (COL-1), as well as enhanced the expression of Janus kinase (JAK) and signal transducer and activators of transcription (STAT) pathway-related proteins, p-JAK1, p-JAK2, p-STAT1, and p-STAT3, in HK-2 cells cocultured with macrophages [167]. Additionally, the administration of anti-miR-214 before UUO was associated with antifibrotic effects. Anti-miR-214 used in that experiment did not block the activation of Smad2/3. Moreover, the inhibition of TGF-β accompanied by the deletion of miR-214 was associated with additional renal protection. The inhibition of canonical TGF-β signaling failed to affect the regulation of endogenous miR-214. All the aforementioned findings provide evidence for a Smad2/3-independent profibrotic effect of miR-214 [108].

In another study, the administration of anti-miR-21 oligonucleotides to mice with Alport disease decreased the rate of its progression, hampered interstitial fibrosis and glomerulosclerosis, limited tubular injury and inflammation, as well as improved survival [90].

The clinical application of miRNA-based medicinal products in humans is still very challenging. The translation of the results obtained in preclinical studies is associated with some obstacles due to the fact that the miRNAs are not regulated in a cell-type or organ characteristic manner; therefore, the targeted modulation of their expression may induce changes in other, unrelated organs [162]. Thus, targeting miRNAs to the kidney is still challenging, and as it can potentially trigger unwanted effects in other tissues and organs, only a sparse number of human trials have been conducted [163]. To circumvent the problem of off-target effects, local injections to specific tissues or even cells may be used. Nanoparticles coated with antigens recognizable by specific receptors expressed on the targeted cells have been engineered; however, the preliminary tests revealed problems with their efficient internalization in cells and the release of antagomirs into the cytoplasm, which shows that this technique still requires optimization [168]. Moreover, a single miR can regulate several mRNA targets, but it can also be regulated by other miRs [169]. Therefore, in case of the administration of miRs with mimics or antagomirs, the aforementioned off-target effects could be more pronounced compared to standard drugs. Furthermore, the level of miRNAs, the response to therapy, and the efficiency can be affected by age, gender, and ethnicity, the use of some medications, or comorbidities. The clinical evidence has shown enhanced pharmacological effects of antagomirs in the presence of some disease conditions [170].

As a result, only a few miRNA-based RNA inhibitors have undergone tests in humans. Since administration of anti-miR-21 oligonucleotides was found to be beneficial and safe in animal models, the safety of RG-012, an antagomir modified with 2′-fluoro substitutions (PS) and 2′-O-methyl phosphorothioate (2-O-MOE), has been assessed in patients with Alport syndrome [77,171]. However, the results of this clinical trial have not been disclosed. Anti-miR-21 (Lademirsen; SAR339375) was further studied in a phase II clinical trial in patients with hereditary nephritis (Alport syndrome); however, this study was terminated since the results of the futility analysis were not satisfactory.

## 7. Conclusions

The finding that miRNAs act as vital regulators of various pathophysiological processes triggered the need to use them as therapeutic agents in various diseases. Since renal fibrosis poses an important problem in chronic kidney disease, many scientists have focused on the determination of miRNAs associated with kidney fibrosis. In this review, we presented the role of several miRNA in renal fibrosis and potential pathways involved. However, as well as those mentioned here, other miRs have also been suggested to play a role in this process in CKD. The conflicting results of some of the studies described above could be because the expression and regulation of miRNAs occur in a tissue- and even cell-dependent manner. Single miRs may regulate several mRNA targets, but at the same time, they could also be regulated by other miRs. The importance of miRs in a clinical setting requires the validation of the obtained results in large studies and clinical trials. Since miRNAs have great potential, their analysis may improve the diagnostic and therapeutic strategies.

## Figures and Tables

**Table 1:** Roles of described miRNA in renal fibrosis.

miRNA	Pathways/Targets	Effect
		PROFIBROTIC
miR-21	PPAR-α axis [77] PTEN/Akt pathway [61] ERK signaling (ERK1/2 and TGF-β/Smad7 pathways) [82] HIF-1α/VEGF pathway IKKβ/mTORC1	- Regulation of fibroblast proliferation [61,71,72] - Impact on the expression of several metalloproteinases (MMPs) [79] - Downregulation of Mpv17-like protein, which regulates ROS [77,81] - Reduction of DDAH1 activity/expression [89] - Promotion of EMT and ECM deposition through Smad7 - Impact on lipid oxidation and metabolism pathway
miR-34a	TGF-β1/Smad signal pathway PI3K/Akt signaling pathway Downregulation of Klotho [39] Notch1 Inhibition of Bcl-2 SIRT1 PPAR-γ, PNUTS, RXRα, Nectin-1, and ABCA3	- Acceleration of profibrotic phenotype transition in tubular epithelial cells and increased proliferation of fibroblasts [93,97] - Promotion of EMT [17] - Regulation of apoptosis, autophagy, and cellular senescence in epithelial/endothelial cells and fibroblasts
miR-130a-3p	TGF-β1/Smad pathway Target: SnoN [35]	- Modulation of EMT and fibrosis [35]
miR-199a	HIF-1α TWIST transcription factor [104]	- Promotion of hypoxic state via HIF-1α, and the activation of the TWIST transcription factor [104]
miR-214	PI3K/Akt signaling pathway PTEN [109]	- TGF-β1-related enhancement of EMT - Activation of PI3K/Akt signaling pathway via downregulation of targeted PTEN [109] - Promotion of TGF-β1-induced tubulointerstitial interstitial transformation and renal interstitial fibrosis
miR-433	TGF-β/Smad3-Azin1 signaling pathway [112]	- Promotion of TGF-β signaling and fibrosis [112]
		ANTIFIBROTIC
miR-29 family (miR-29a, miR-29b, and miR-29c)	Target: collagen I and IV, elastin, integrin-β1, laminins, and fibrillin [124,125] Downstream inhibitor of TGF-β/Smad3-mediated fibrosis [127]	- Decreased expression of approximately 20 various genes related to collagen formation - Suppression of matrix accumulation and promotion of cell death, provided that it is profibrogenic cells that undergo apoptosis [81,128]
miR-27b-3p	Target: pSTAT1 and STAT1 [131] Direct target: TGF-β receptor 1 and SMAD2	- Repression of TGF-β1-induced EMT through the downregulation of α-SMA, fibronectin, collagen III, and vimentin in cell cultures [131] - Limitation of renal fibrosis (in vitro and in vivo) through EMT inhibition - Inhibition of TGF-β1-induced and Fas-mediated apoptosis in human kidney 2 cells through the downregulation of active caspase 3, Fas, and active caspase 8 [131] - Inhibition of fibroblast activation - Reduced expression of pSTAT1 and STAT1 [131]
miR-30 family (miR-30a, -30b, -30c, -30d, and -30e)	Target: SOX9 Downregulates UCP2 [140] Downregulates the expression of CTGF [142] Nectin1	- Limited production of ECM protein by renal tubular epithelial cells - Inhibited phenotypic modifications of ECM via targeting UCP2 [140] - Lowering of collagen I, vimentin, fibronectin, and alpha-smooth-muscle actin (α-SMA) expression [143] - Increased E-cadherin expression [143]
hsa-miR-3607-3p and hsa-miR-4709-3p	mTOR signaling pathway [145] hsa-miR-3607-3p target: ITGB8 hsa-miR-4709-3p target: calmodulin 3	- Regulation of actin cytoskeleton - Changes in intracellular calcium levels [126]
miR-671-5p	Target: TGF-β1 and CTGF	- Increased expression of many fibrosis-related proteins, including collagen I, α-smooth-muscle actin (α-SMA), and fibronectin [147] - Inhibition of miR-671-5p decreased kidney interstitial fibrosis [147]
miR299a-5p	Impact on activin A activity and Smad3 signaling [115,116].	- Limited expression of Zeb1 and Zeb2 expression and antifibrotic effects
		CONTROVERSIAL ROLE
miR-192 family	HNF-1 TGF-β signaling [151] miR-200b/c, Col1a2, Col4a1 [153]	- Downregulation of Zeb1/2 expression to enhance collagen expression and renal fibrosis - In renal mesangial cells, upregulation of TGF-β1 levels by amplifying circuits involving miR-192 or miR-200b/c and TGF-β1 [153] - miR-192 inhibitors reduced the expression of miR-200b/c, Col1a2, Col4a1, and TGF-β1 in mice mesangial cells and the renal cortex [153]
miR-200 family	TGF-β expression ZEB1 and ZEB2	- Maintenance of epithelial cell differentiation [81] - Suppression of TGF-β2 expression, preventing the development of renal fibrosis [157] - Protection of epithelial cells against the action of pro-EMT factors such as TGF-β1 - Promotion of mesenchymal-to-epithelial transition

ABCA3, ATP-binding cassette sub-family A member 3; Akt, protein kinase B; Bcl-2, B-cell CLL/lymphoma 2; CTGF, connective tissue growth factor; DDAH1, dimethylarginine dimethylaminohydrolase 1; ECM, extracellular matrix; EMT, epithelial–mesenchymal transition; ERK, extracellular signal-regulated kinases; HIF-1α, hypoxia-inducible factor 1α; HNF-1, hepatocyte nuclear factor 1; IKKβ, inhibitor of nuclear factor kappa B kinase subunit β; ITGB8, integrin subunit beta 8; Nectin1, nectin cell adhesion molecule 1; Notch1, neurogenic locus notch homolog protein 1; mTOR, mammalian target of rapamycin kinase; mTORC1, mammalian target of rapamycin complex 1 or mechanistic target of rapamycin complex 1; PI3K, phosphoinositide-3-kinase; PNUTS, phosphatase 1 nuclear targeting subunit; PPAR-α, peroxisome proliferator-activated receptor alpha; PPAR-γ, peroxisome proliferator-activated receptor γ; PTEN, phosphatase and tensin homolog deleted on chromosome 10; ROS, reactive oxygen species; RXRα, retinoid X receptor α; SIRT1, Sirtuin 1; Smad7, SMAD family member 7; SnoN, Ski-novel protein; SOX9, SRY-box transcription factor 9; TGF-β, transforming growth factor β; UCP2, mitochondrial uncoupling protein 2; VEGF, vascular endothelial growth factor; ZEB1, Zinc finger E-box binding homeobox 1.

## Data Availability

Not applicable.
